# Case report: CAR-T cell therapy-induced cardiac tamponade

**DOI:** 10.3389/fcvm.2023.1132503

**Published:** 2023-03-20

**Authors:** Sacha Sarfati, Misa Eugène Norbert, Antoine Hérault, Marion Giry, Jade Makké, Maximilien Grall, Arnaud Savouré, Vincent Camus, Mustafa Alani, Fabienne Tamion, Jean-Baptiste Latouche, Christophe Girault

**Affiliations:** ^1^ Medical Intensive Care Unit, Normandie Univ, UNIROUEN, UR 3830, CHU Rouen, Medical Intensive Care Unit, Rouen, France; ^2^ INSERM U1234, University of Rouen Normandie, Rouen, France; ^3^ Department of Clinical Hematology, Centre Henri Becquerel, Rouen, France; ^4^ Department of Cardiology, CHU Rouen, Rouen, France; ^5^ Department of Hematology and INSERM U1245, Centre Henri Becquerel, Rouen, France; ^6^ INSERM U1096, Normandie Univ, UNIROUEN, CHU Rouen, Medical Intensive Care Unit, Rouen, France; ^7^ INSERM U1245, Normandie Univ, UNIROUEN, Institute for Research and Innovation in Biomedecine (IRIB), Rouen, France

**Keywords:** CAR-T cell, pericarditis, cardiac tamponade, pericardial effusion, CRS, ICU, case report

## Abstract

CD19-specific chimeric antigen receptor T (CAR-T) cell therapy has recently been shown to improve the prognosis of refractory diffuse large B-cell lymphoma (DLBCL). However, CAR-T cells may induce numerous adverse events, in particular cytokine release syndrome (CRS) which is frequently associated with cardiovascular manifestations. Among the latter, acute pericardial effusion represents less than 1% of cases and cardiac tamponade has only been reported once. The management and outcome of these severe complications are not well established. We report here, a case of cardiac tamponade associated with CRS in a context of CAR-T cell therapy, which required urgent pericardiocentesis.

**Case summary:**

A 65-year-old man with refractory DLBCL was treated with CAR-T cell therapy. He had a history of dilated cardiomyopathy with preserved ejection fraction and transient atrial fibrillation. A pericardial localization of the lymphoma was observed on the second relapse. One day after CAR-T cell infusion the patient was diagnosed with grade 1 CRS. Due to hypotension, he was treated with tocilizumab and dexamethasone, and then transferred to intensive care unit (ICU). Echocardiography performed at ICU admission showed acute pericardial effusion with signs of right ventricular heart failure due to cardiac tamponade. It was decided to perform pericardiocentesis despite grade IV thrombocytopenia in a context of aplasia. Analysis of pericardial fluid showed a large number of lymphoma cells and 73% of CAR-T cells amongst lymphocytes, a level that was similar in blood. Hemodynamic status improved after pericardiocentesis, and no recurrence of pericardial effusion was observed. The presence of a high count of activated CAR-T cells in the pericardial fluid as well as the short interval between CAR-T cells injection and the symptoms appear as potential arguments for a direct action of CAR-T cells in the mechanism of this adverse event. The patient was discharged from ICU after two days and initially exhibited a good response to DLBCL treatment. Unfortunately, he died fifty days after starting CAR-T cell therapy due to a new DLBCL relapse.

**Conclusion:**

Patients with a pericardial localization of DLBCL should be assessed for a risk of cardiac tamponade if receiving CAR-T cell therapy and presenting CRS. In this case, cardiac tamponade seems directly related to CAR-T cell expansion. Pericardiocentesis should be considered as a feasible and effective treatment if the risk of bleeding is well controlled, in association with anti-IL6 and corticosteroids.

## 1. Introduction

Among novel cancer therapies, CD19-specific chimeric antigen receptor T (CAR-T) cell therapy is one of the most successful and promising treatments for refractory hematologic malignancies, in particular aggressive diffuse large B-cell lymphoma (DLBCL) (1). However, CAR-T cells may cause numerous and potentially severe adverse events (2), notably cytokine release syndrome (CRS) and immune effector cell-associated neurotoxicity syndrome (ICANS). While ICANS is characterized by altered mental status, the main symptoms of CRS are fever and hypotension that can lead to shock, multiple organ failure and even death (3). A wider use of CAR-T cell therapy has led to an increase in the number of reports of cardiovascular adverse events (CVAE), including heart failure, arrhythmia and pericardial effusion. These CVAE are often associated with CRS, but are sometimes reported as an independent entity as well (4–8). In this group, pericardial complications account for less than 1% of all CVAE and are therefore very little known (6). Furthermore, to our knowledge, only one case of cardiac tamponade with CAR-T cell-associated CRS has been reported (9). Therefore, we report here a case of CAR-T cell-induced CRS complicated with acute pericardial effusion and cardiac tamponade requiring urgent pericardiocentesis.

## 2. Case description

A 65-year-old man was diagnosed with DLBCL, Ann Arbor Stage IV. He had a history of dilated cardiomyopathy with preserved ejection fraction, transient atrial fibrillation following a rituximab injection, type 2 diabetes, class III obesity, and localized melanoma with complete exeresis more than fifteen years before.

Echocardiography performed before DLBCL treatment showed signs of known dilated cardiomyopathy with good left and right ventricular function and no pericardial effusion.

**Figure 1:**
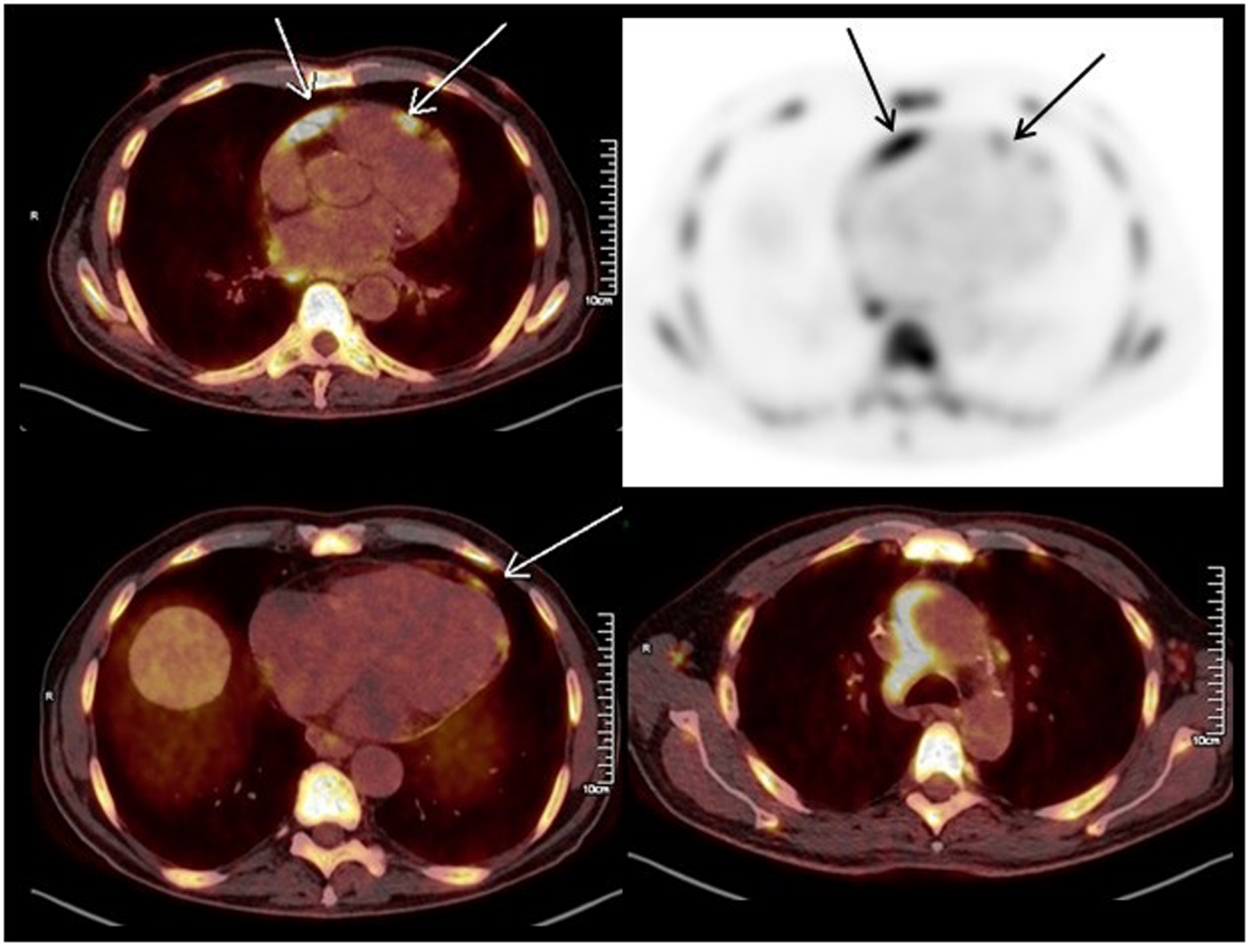
Positron emission tomography-computed tomography performed before CAR-T cell therapy showing multiple lymphoma lesions including in the pericardium (arrows).

He received first-line chemotherapy with rituximab, cyclophosphamide, doxorubicin, vincristine, prednisolone, and methotrexate but exhibited no metabolic response on positron emission tomography-computed tomography (PET-scan). Therefore, second-line chemotherapy was initiated with rituximab, dexamethasone, cytarabine and cisplatin, which achieved a complete metabolic response. Then, the patient received an autologous stem cell transplant conditioned with carmustine, etoposide, cytarabine and melphalan. Less than one year later, the patient presented abdominal pain, leading to a transjugular hepatic biopsy showing a stage IV DLBCL relapse. It was then decided to perform CAR-T cell therapy. At this time a PET-scan found systemic multiple lymphoma localizations with hepato-splenic, bone, abdominal and thoracic lesions as well as pericardial hypermetabolism and a small aortic cross hematoma (Figure 1). Rituximab, ifosfamide, carboplatin and etoposide were administered as a bridge to CAR-T cell therapy with good metabolic response. Due to a transient, asymptomatic COVID-19 infection, CAR-T cell reinjection was delayed for one month. Thirteen days before reinjection of CAR-T cells, a PET-scan showed progressive DLCBL and persistent pericardial hypermetabolism.

**Figure 2:**
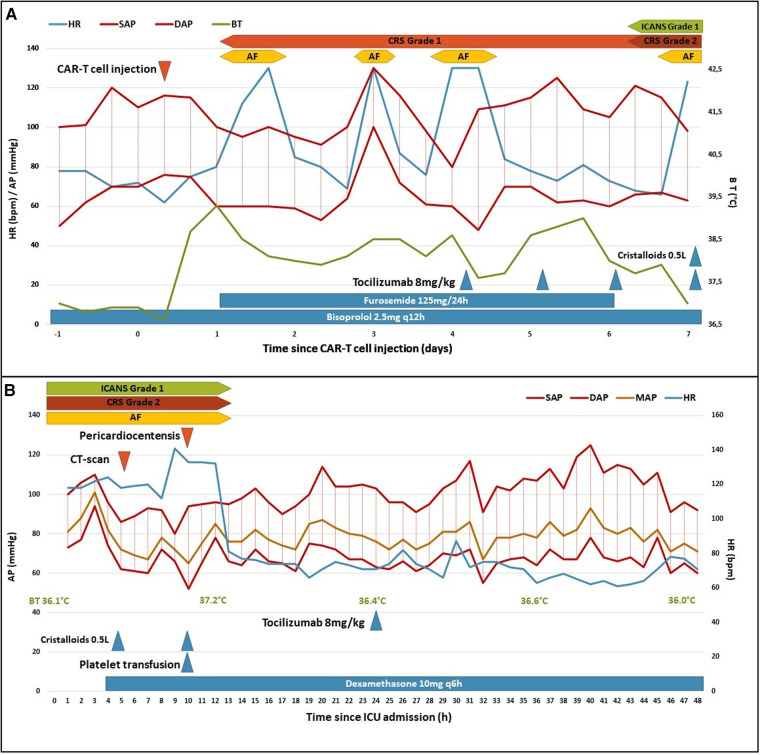
Clinical course following CAR-T cell injection (**A**) and ICU admission (**B**). AF, atrial fibrillation; AP, arterial pressure; BT, body temperature; CRS, cytokine release syndrome; DAP, diastolic AP; HR, heart rate; ICANS, immune effector cell-associated neurotoxicity syndrome; ICU, intensive care unit; MAP, mean AP; SAP, systolic AP.

The patient was referred to our hematology department to receive CAR-T cell therapy with axicabtagene ciloleucel. At admission, cardiac examination was normal, and an electrocardiogram (ECG) showed normal sinus rhythm with known right bundle branch block and no other abnormalities. The patient presented chemotherapy-induced myelosuppression and tumor lysis syndrome. Following lymphodepleting chemotherapy with cyclophosphamide and fludarabine, CAR-T cells were administered (0.65 × 10^8^ cells/kg). The patient developed fever (≥38°C) on the day of CAR-T cell infusion (day 0) associated with atrial fibrillation and was then diagnosed with grade 1 CRS according to the American Society for Transplantation and Cellular Therapy grading of CRS (3). Empiric administration of antibiotics was initiated with piperacillin-tazobactam and teicoplanin. On day 1, considering atrial fibrillation, low urine output and clinical fluid overload, furosemide was initiated. On day 4, due to persistence of fever, tocilizumab (8 mg/kg/day) was initiated (Figure 2A). Clinically, fluid overload increased, and atrial fibrillation was intermittent from day 1 to day 6. On day 7, the patient presented hypotension [grade 2 CRS (3)] and delirium causing suspicion of grade 1 ICANS (3). In this context, a volume expansion of 0.5 L of crystalloids was administered, dexamethasone was initiated, and the patient was transferred to the intensive care unit (ICU).

**Figure 3:**
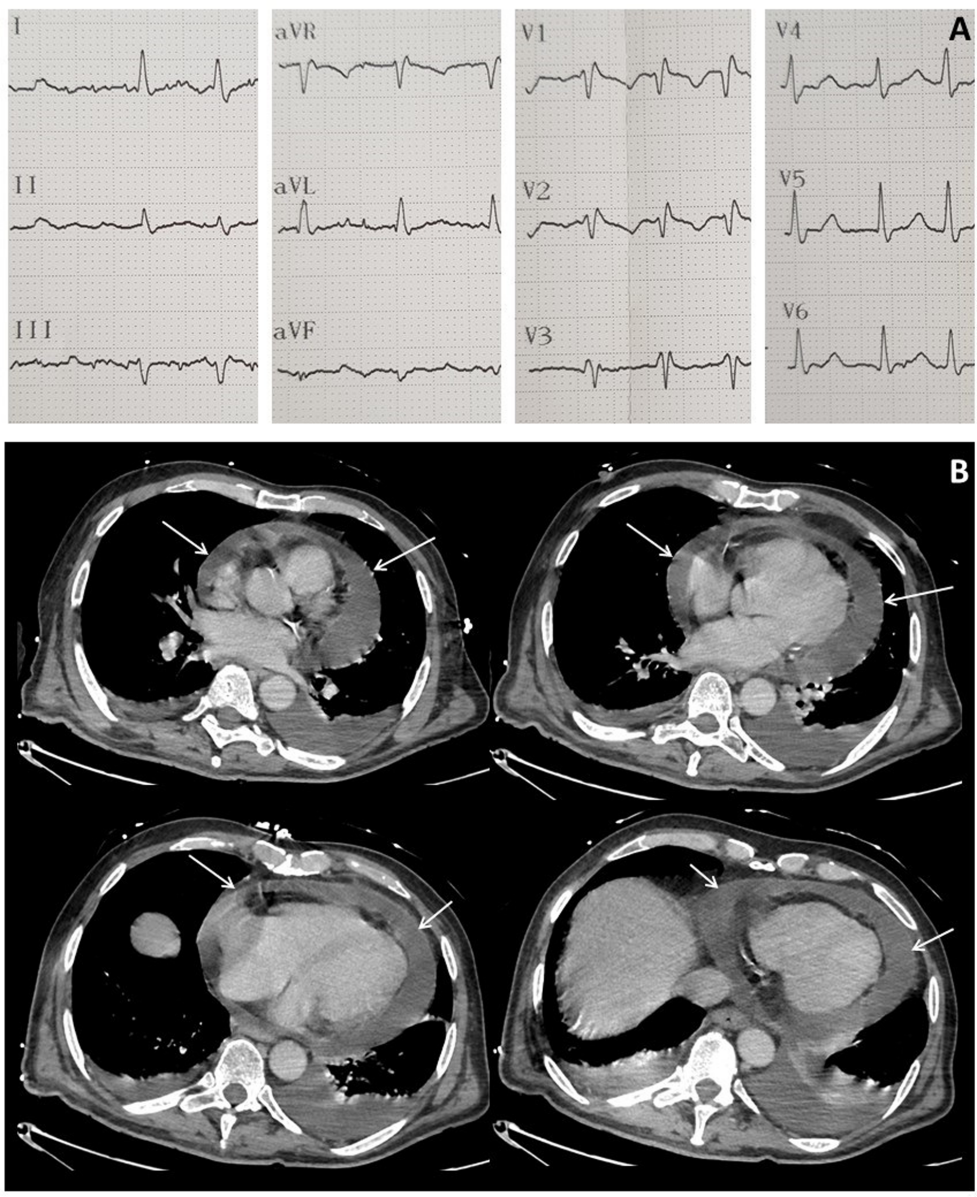
Electrocardiogram at ICU admission showing atrial fibrillation and right bundle branch block (**A**) and computed tomography performed after CAR-T cell therapy showing major circumferential pericardial effusion (arrows) (**B**).

At admission to ICU, his blood pressure was 109/80 mmHg, heart rate was 118 beats/min, body temperature was 36.1°C, respiratory rate was 35 cycles/min and percutaneous oxygen saturation (SpO_2_) was 95% in air. The time course of the vital signs and medications following ICU admission is shown in Figure 2B. Edema of the lower limbs was observed, but no other sign of right ventricular dysfunction was noted. Heart sounds were decreased but no pericardial friction was heard. Serum C-reactive protein (CRP) was moderately elevated to 15 mg/L (normal range: ≤5 mg/L) as well as N-Terminal pro-Brain Natriuretic Peptide (3171 ng/L; normal range: ≤376 ng/L) but serum troponin T was normal (13 ng/L; normal range: ≤14 ng/L). An ECG showed atrial fibrillation, right bundle branch block but no sign of pericarditis (Figure 3A).

In a context of severe thrombocytopenia (26,000/µL), brain and chest CT-scans were performed to investigate the sudden delirium and to monitor the aortic cross hematoma. No intracranial or thoracic bleeding was detected, but an increase in pericardial effusion was noted (Figure 3B).

Echocardiography revealed decreased right ventricular function (TAPSE = 15 mm) with marked pericardial effusion and compression on the right atrium collapsed in the early diastolic phase. Left ventricular function was moderately decreased (LVEF = 45%). A distended inferior vena cava with a reduced respiratory diameter was observed. Based on these findings, cardiac tamponade was diagnosed. After platelet transfusion and a volume expansion of 0.5 L of crystalloids, it was then decided to perform immediate pericardiocentesis, which removed 300 ml of hematic fluid.

After pericardiocentesis, the patient's hemodynamic status improved: atrial fibrillation stopped within the next hour, and arterial blood pressure remained stable. Delirium resolved the following day and no more fever was reported. Two days after (day 9), the patient was discharged from ICU and transferred back to hematology department. Control echocardiography before transfer showed no recurrence of pericardial effusion and good biventricular systolic function.

**Figure 4:**
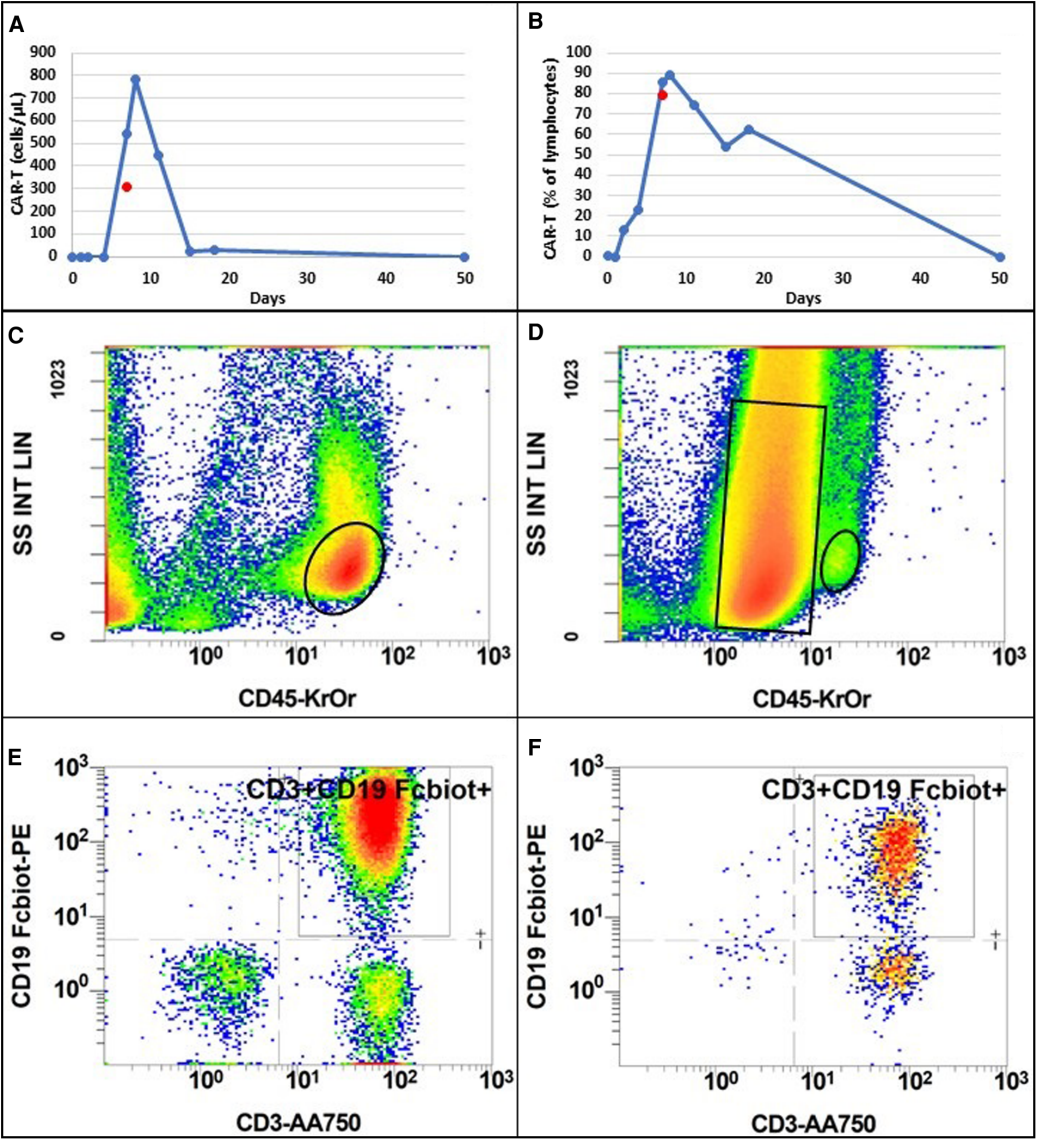
Evolution of CAR-T cell absolute number (**A**) and percentage of total lymphocytes (**B**) following injection, in blood (blue dots and line) and in pericardial fluid (red dot). Flow cytometry analysis of blood (**C**) and pericardial fluid (**D**) according to size (SS INT LIN) and CD45 expression (CD45-KrOr). Black ovals represent lymphocyte population. The black rectangle represents lymphoma population only present in pericardial fluid. Among lymphocyte population, CAR-T cells were selected according to CD3 and CD19 Fc-biotine expression in blood (**E**) and pericardial fluid (**F**). Colors from blue to red indicate an increase in cell count.

CAR-T cell expansion was monitored with regular measurement in blood. Analysis was performed in flow-cytometry. Evolution of CAR-T cell expansion was similar to that in most patients with a peak at around day 10 after reinjection (Figures 4A,B). Detection of CAR-T cells was based on size, and CD45+ CD3+ CD19+ FCbiot+ cell expression (Figures 4C–F). Pericardial fluid analysis, performed with the same method, showed 26,841 cells/µl, of which 98% were lymphoma cells (small size CD45- CD19+ CD3- CD56+ DR+) and 2% were lymphocytes (CD45+), of which 72.8% (310 cells/µL) were CAR-T cells with more than 80% of activated cells (DR+) (Figures 4D,F). The concomitant analysis of blood showed no lymphoma cells and 880/µL of lymphocytes of which 88.7% were CAR-T cells with more than 90% of activated cells (Figures 4C,E). In blood, detection of CAR-T cells decreased rapidly after day 15 (Figures 4A,B).

In hematology ward, dexamethasone was discontinued on day 14 and the patient presented no new fever until day 33, concomitant with *Pseudomonas aeruginosa* bacteremia and cervico-facial cellulitis. A PET-scan on day 30 showed a complete metabolic response and total regression of the pericardial localization. There was no recurrence of CVAE. The cellulitis improved from day 40 but myelosuppression persisted. On day 49, the patient developed abdominal pain. A CT-scan revealed a major relapse of DLCBL lesions with hepato-splenic localizations. In this context, the patient received best supportive care and died fifty days after starting CAR-T cell therapy.

## 3. Discussion

CAR-T cell therapy has allowed a major improvement in the management and prognosis of refractory DLBCL, previously with extremely poor outcomes. Clinical trials have shown a high rate of complete response, and real-world evidence confirms these findings (2). The growing use of CAR-T cell therapy has also led to enhanced reporting of adverse events (10). CRS and ICANS havealready been described in clinical trials, allowing more reports of CVAE more recently (4). The diversity of presentation of CVAE and a frequent association with CRS have made it difficult to identify them as an independent entity (11). However, it remains unclear whether the cause of CVAE is linked to a direct action of CAR-T cells or whether it is driven by systemic aggression (12).

Among CVAE associated with CAR-T cell therapy, pericarditis events remain uncommon. Pericarditis was described as representing 0.4% of all CVAE, mostly associated with CRS (6). To our knowledge, cardiac tamponade in association with CRS following CAR-T cell therapy has only been reported once. Indeed, Moriyama et al. (9) reported the case of a 59-year-old man with refractory DLBCL who was also treated with CAR-T cell therapy and who presented cardiac tamponade associated with grade 4 CRS. However, these authors did not perform pericardiocentesis in their patient who had severe myelosuppression and shock, which they considered a major risk factor for complications (9). The management of their patient, who died 6 months after starting CAR-T cell therapy, was based on anti-inflammatory therapy with tocilizumab and corticosteroids and hemodynamic support as recommended for severe CRS (13).

In our case, the patient was initially diagnosed with CRS based on an association of fever and hypotension. The appearance soon after of delirium led to suspicion of concomitant ICANS. However, all these symptoms resolved quickly after pericardiocentesis and, in theory, all of them can be attributed to pericardial effusion with cardiac tamponade. At this stage, it remains unclear whether the tamponade was caused by systemic inflammation (CRS), increasing capillary permeability, and eventually worsening the existing pericardial effusion, or if it was related to a direct infiltration of CAR-T cells in an important localization of the disease. The presence in the pericardial fluid of a high count of activated CAR-T cells as well as the clinical presentation with a rapid resolution after pericardiocentesis may favor the second hypothesis. Nevertheless, as the patient received treatment for CRS as well, it is difficult to consider that CRS played no role in the clinical presentation. Furthermore, the tamponade occurred soon after the peak of CAR-T cell expansion, and the percentage of CAR-T cells among lymphocytes was similar in pericardial fluid and blood (Figures 4A,B).

The direct toxicity of engineered CAR-T cells on cardiac tissue has already been described (14), but the strong association of CVAE with CRS following CAR-T cell therapy suggests that a nonspecific form of cytokine-associated cardiotoxicity may be at play (15).

One important finding from our case report and that of Moriyama et al. (9), is that both patients initially presented a pericardial localization of the lymphoma. Therefore, we should consider a pre-existing pericardial localization of the lymphoma as a major risk factor for CAR-T cell therapy-induced pericardial effusion with cardiac tamponade. However, large prospective cohort studies will be necessary to assess the risk factors associated with this major CVAE.

Our patient presented only moderate hemodynamic failure with no need for vasopressors. Unlike the case reported by Moriyama et al. (9), we decided to perform pericardiocentesis as recommended for cardiac tamponade (16), which led to rapid improvement of the patient's clinical status. Indeed, the risk of intra-pericardial bleeding due to severe thrombocytopenia and, therefore, of increasing the tamponade exposing to the risk of severe cardiogenic shock, were considered as strong arguments for performing rapid pericardiocentesis. In addition, the risk of complications related to pericardiocentesis, mainly major bleeding in the present case due to severe thrombocytopenia was minimized by platelet transfusion before the procedure, echocardiographic guidance and an experienced cardiologist who performed pericardiocentesis. In fact, it has been shown that percutaneous pericardiocentesis can be performed safely in cancer patients with severe thrombocytopenia by following a rigorous technical approach (17). In the previous case (9), interestingly, anti-inflammatory therapy (tocilizumab and high dose corticosteroids) and appropriate management of hemodynamic failure were able to resolve the tamponade within one week without pericardiocentesis. Finally, based on the present case and that of Moriyama et al. (9), CAR-T cell therapy-induced pericardial effusion with cardiac tamponade requires prompt anti-inflammatory therapy in all cases. Pericardiocentesis appears feasible, allows a rapid resolution of this severe CVAE, and should be discussed immediately before an irreversible worsening, considering the benefit-risk balance for the patient (17, 18).

## 4. Conclusion

We report the case of a 65-year-old man with refractory stage IV DLBCL with a pericardial localization treated with CAR-T cell therapy complicated by severe CVAE, i.e., acute pericardial effusion with cardiac tamponade associated with grade 2 CRS. Anti-inflammatory therapy for CRS associated with pericardiocentesis rapidly resolved the symptoms and allowed a timely discharge from ICU. This severe CVAE could be related to CAR-T cell-associated CRS or a direct infiltration of CAR-T cells in a pericardial localization of DLBCL. In our case, the presence of activated CAR-T cells in the pericardial fluid could favor a direct action of CAR-T cells. When CRS occurs following CAR-T cell therapy in a patient with a pre-existing pericardial localization of DLBCL, CVAE including cardiac tamponade should be considered and echocardiography should be performed immediately. Although anti-inflammatory therapy remains the cornerstone of CRS management, given the uncertainty about the pathophysiology of CAR-T cell-induced pericardial effusion with cardiac tamponade, pericardiocentesis should also be considered before irreversible cardiogenic shock according to the benefit-risk balance for the patient.

## Data Availability

The raw data supporting the conclusions of this article will be made available by the authors, without undue reservation.
